# Clinical and genetic characteristics of glucose transporter 1 deficiency syndrome in a large cohort of Chinese patients

**DOI:** 10.1007/s12519-025-00884-9

**Published:** 2025-03-06

**Authors:** Mei-Jiao Zhang, Shi-Min Zhang, Qing-Ping Zhang, Yong-Xin Wen, Jia-Ping Wang, Yu-Wu Jiang, Xin-Hua Bao

**Affiliations:** 1 Department of Pediatrics, Peking University First Hospital, Beijing, China; 2 Department of Pediatrics, Peking University People’s Hospital, Beijing, China; 3 Department of Pediatric Neurology, Guangdong Women and Children Hospital, Guangdong, China; 4 Department of Neurology, Beijing Children′S Hospital, Capital Medical University, Beijing, China

**Keywords:** Epilepsy, Glut1DS, Movement disorders, *SLC2A1*

## Abstract

**Background:**

Mutations in the *SLC2A1* gene cause glucose transporter type 1 deficiency syndrome (Glut1DS). This study aimed to investigate the clinical and molecular genetics characteristics of Chinese patients with Glut1DS.

**Methods:**

The clinical data of patients with Glut1DS were analyzed retrospectively. *SLC2A1* mutation analysis was performed using Sanger sequencing or next-generation sequencing (NGS). Multiplex ligation-dependent probe amplification (MLPA) was conducted in patients with negative results.

**Results:**

A total of 90 patients were diagnosed with Glut1DS, including 63 (70%) classic type and 27 (30%) non-classic type. Seizures occurred in 69 patients (77%), movement disorders were observed in 58 (68%), and episodic eye–head movements were noted in 17 (19%). Cerebrospinal fluid (CSF) glucose levels were available for 73 patients (81%), ranging from 1.0 to 2.6 mmol/L (median 1.9 mmol/L), with 90% (66/73) of patients showing levels below 2.2 mmol/L. Additionally, CSF-to-blood glucose ratios measured in 71 patients (79%) ranged from 0.20 to 0.63 (median 0.37), with 87% (62/71) of patients having ratios below 0.45. Genetic analysis identified 69 variants of the *SLC2A1* gene including 39 previously reported and 30 unreported variants. The two most common variants were c.997C > T (p.Arg333Trp) and c.988C > T (p.Arg330*). Following ketogenic diet therapy, seizures were controlled in 47 of 57 patients (82%), movement disorders resolved in 18 of 47 patients (38%), and improved in 26 of 47 patients (55%).

**Conclusions:**

The clinical manifestations of Glut1DS primarily include seizures, movement disorders, and developmental delay. Most affected children had CSF glucose levels below 2.2 mmol/L, with CSF-to-blood glucose ratios under 0.45. Two of the most common *SLC2A1* variants were identified in our cohort. Ketogenic diet therapy was effective in controlling seizures, improving movement disorders, and was well tolerated.

**Graphical abstract:**

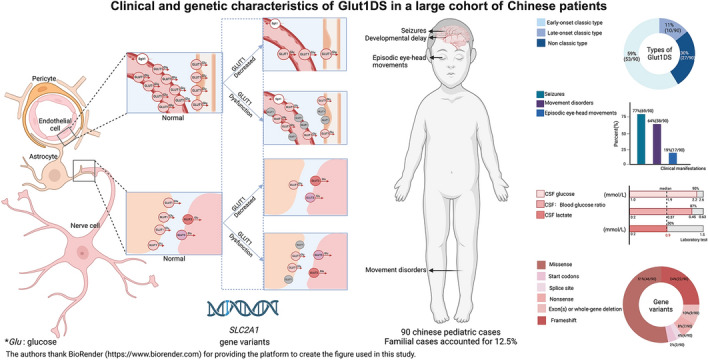

**Supplementary Information:**

The online version contains supplementary material available at 10.1007/s12519-025-00884-9.

## Introduction

The *SLC2A1 gene*, located on chromosome 1p34.2, encodes glucose transporter protein 1 (GLUT1), which facilitates glucose transport across the blood–brain barrier into neurons and glial cells. Mutations in *SLC2A1* result in either dysfunction or reduced expression of GLUT1, impairing glucose delivery to the brain. This condition, known as GLUT1 deficiency syndrome (Glut1DS), was first described by De Vivo in 1991 [1]. Characteristic symptoms include early onset drug-resistant epilepsy, developmental delay, spasticity, ataxia, and dystonia. Dysarthria and speech impairment are also significant clinical features of Glut1DS. To date, over 500 cases have been reported globally [2]. The first Chinese case, diagnosed by the Department of Pediatrics at Peking University First Hospital, was reported in 2012 [3]. The phenotypic spectrum of Glut1DS has expanded to include paroxysmal exercise-induced dyskinesia (PED), complex movement disorders, and intellectual disability. Prevalence estimates range from 1.65 to 4.13 per 100,000 live births in Europe and North America [4, 5]. Low cerebrospinal fluid (CSF) glucose levels with normal blood glucose levels are key diagnostic markers, and pathogenic *SLC2A1* variants confirm the diagnosis. Ketogenic diet therapy is the first-line intervention for Glut1DS, significantly controlling seizures, improving motor and cognitive impairments, and preventing irreversible brain damage from delayed treatment. However, due to its rarity, complex presentations, and potential for false-negative genetic results, diagnosing Glut1DS remains challenging. In China, limited research [6, 7] on Glut1DS has contributed to insufficient awareness and a lack of standardized diagnostic and treatment guidelines. This study aims to summarize the clinical and genetic characteristics of Chinese Glut1DS patients and evaluate the outcomes and side effects of ketogenic diet therapy.

## Methods

### Study population

Patients diagnosed with Glut1DS at Peking University First Hospital from November 2008 to October 2024 were included in this study. Selection criteria were based on the 2020 recommendations of the international Glut1DS study group [8]. Inclusion criteria encompassed: (1) clinical manifestations, such as seizures, episodic or persistent movement disorders triggered by fasting, fatigue, or physical activity, and developmental delays in motor and intellectual abilities; (2) low CSF glucose levels (< 2.8 mmol/L) with a CSF-to-blood glucose ratio < 0.6; and (3) pathogenic *SLC2A1* variants identified through genetic analysis.

Exclusion criteria included increased CSF white blood cell count, protein, or lactate levels; conditions causing reduced CSF glucose; and *SLC2A1* variants classified as benign or likely benign according to the American College of Medical Genetics and Genomics (ACMG) guidelines.

Diagnosis required fulfillment of criteria (1), (2), and (3), or criteria (1) and (3). Clinical diagnosis was permissible with negative or absent *SLC2A1* testing, provided that criteria (1) and (2) were met and exclusion criteria satisfied.

Microcephaly is defined as a head circumference that is more than 2 standard deviations (SD) below the mean for age and sex. A smaller head circumference is defined as one that falls between 1 and 2 SD below the mean for age and sex.

### Genetic analysis

Genomic deoxyribonucleic acid (DNA) was extracted from peripheral blood lymphocytes. Before 2013, all *SLC2A1* exons were amplified using polymerase chain reaction (PCR) and directly sequenced for 18 cases and their parents. From 2013 onward, next-generation sequencing (NGS) was employed for an additional 72 patients. Variants detected via NGS were confirmed by Sanger sequencing in probands and some family members. If pathogenic variants were not identified, multiplex ligation-dependent probe amplification (MLPA) or low-depth whole-genome copy-number variation sequencing (CNV-Seq) was performed to detect large deletions or duplications. Variants were classified according to ACMG guidelines using population frequency data, in silico predictive tools, and locus-specific databases. For variants of uncertain significance rated by ACMG, the protein structure visualization software pMOL was used to draw the protein structures of both the wild type and variant to determine the effect of gene variants on protein function.

### Statistical analysis

SPSS software 26.0 version (IBM-SPSS, Chicago, Illinois, USA) was used for data analysis. Normally distributed variables were presented as mean ± SD, and non-normal variables as median with interquartile range (IQR). Categorical variables were reported as frequency (percentage). Continuous variables were compared using the rank-sum test, while categorical data were analyzed using Chi-square or Fisher's exact tests. A *P* value < 0.05 indicated statistical significance.

## Results

A total of 90 patients were included in this study. Among them, 69 met all three diagnostic criteria for Glut1DS, while 17 fulfilled criteria (1) and (3). Four patients met criteria (1) and (2), with all exclusion criteria satisfied, and carried *SLC2A1* variants classified as uncertain significance according to the ACMG guidelines.

### Clinical features

Detailed clinical information is summarized in Tables S1 and Table 1. The cohort included one mother–child pair and one pair of monozygotic twins, representing 88 families. The male-to-female ratio was 1.2:1, with 49 males (54%) and 41 females (46%). Sixty-three patients (70%) presented with the classic type of Glut1DS, comprising 53 early onset and 10 late-onset patients. The remaining 27 patients (30%) exhibited the non-classic type of the disease. Seizures as the initial symptom presented in 41 patients (46%), developmental delay in 36 (40%), persistent or episodic movement disorders in seven (8%), episodic eye–head movements in five (6%), and episodic psychiatric or behavioral abnormalities in one (1%).

**Table 1:** Comparison of clinical findings in Glut1DS

Characteristics	Total (*n* = 90)	Early onset classic phenotype (*n* = 53)	Late-onset classic phenotype (*n* = 10)	Non-classic phenotype (*n* = 27)	*P* value
Age of onset (mon), median(IQR))	8 (3,21)	3 (2,10)	37 (30,46)	16 (8,36)	< 0.001*
Age at medical treatment (mon), median(IQR)	17 (4,36)	5 (2,10)	42 (31,47)	36 (21,47)	< 0.001*
Age of lumbar puncture (mon), median(IQR)	48 (21,68)	27 (6,60)	64 (51,110)	54 (33,77)	0.002*
Age at diagnosis (mon), median (IQR)	44 (21,68)	30 (10,60)	63 (49,110)	51 (28,77)	0.001*
Movement disorders No (%) Yes (%)	32 (36) 58 (64)	23 (43) 30 (57)	5 (50) 5 (50)	4 (15) 23 (85)	0.025*
Episodic psychiatric or behavioral abnormalities No (%) Yes (%)	73 (81) 17 (19)	48 (91) 5 (9)	8 (80) 2 (20)	17 (63) 10 (37)	0.012*
CSF glucose mmol/L, median(IQR)	1.9 (1.7,2.0)	1.8 (1.5,1.9)	2.1 (1.8,2.2)	1.9 (1.8,2.1)	0.003*
Blood glucose, median(IQR)	4.9 (4.6,5.3)	5.0 (4.7,5.4)	4.8 (4.8,4.9)	4.9 (4.6,5.2)	0.317
CSF: blood glucose ratio, median(IQR)	0.37 (0.33,0.42)	0.35 (0.30,0.38)	0.43 (0.41,0.44)	0.39 (0.35,0.42)	0.001*
CSF lactate, median(IQR)	0.9 (0.8,1.0)	0.9 (0.8,1.1)	0.9 (0.8,1.0)	0.9 (0.8,1.1)	0.938
Type of mutations (%) Frameshift, nonsense, cut site, start codon mutation Missense Single, multiple exons, or whole-gene deletion	35 (39) 46 (51) 9 (10)	23 (43) 24 (45) 6 (12)	1 (10) 9 (90) 0 (0)	11 (41) 13 (48) 3 (11)	0.172

*Indicating an overall difference

During the course of the disease, seizures occurred in 69 patients (77%), with onset ages ranging from 1 to 144 months (median: 30 months). Single and multiple seizure types were observed in 44 and 25 patients, respectively, including generalized tonic–clonic seizures in 18 , myoclonic seizures in 11 , absence seizures in nine , atonic seizures in five , generalized tonic seizures in four , myoclonic–atonic seizures in three , and eyelid myoclonia in two . Additionally, focal seizures were reported in 47 patients and status epilepticus in 5 .

Movement disorders were observed in 58 patients (64%, 58/90), with onset ages ranging from 1 to 14 years (median 2 years). The most common movement disorder was ataxia (39 patients), followed by ataxia-spastic gait (17), dystonia (nine ), and PED (eight ). Among these, 21 patients experienced persistent movement disorders with periodic exacerbations, while 20 had episodic movement disorders including PED in 8, ataxia in 12, and dystonia in 3, lasting 20 minutes to two hours. The most common trigger for PED was prolonged walking, whereas fasting was the most frequent trigger for ataxia and dystonia, followed by physical exertion, infections, and emotional excitement. Seventeen patients exhibited persistent movement disorders including ataxia, spastic ataxia, and dystonia, without episodic exacerbations. Progression of movement disorders was observed in 25 patients who were not on a ketogenic diet or a pre-ketogenic diet, with worsening between 2 and 18 years of age (median: 4 years). By October 2024, four patients without a ketogenic diet exhibited resolution of movement disorders, with resolution ages ranging from 10 to 20 years (median: 19 years).

Episodic eye–head movements were noted in 17 patients (19%, 17/90), characterized by rapid multidirectional eye movements with concurrent head movement in the same direction. These episodes began between 2 months and 1.5 years of age (median 4 months), with 71% (12/17) occurring within the first 6 months of life. Symptoms resolved between 1 and 4 years of age (median 2.5 years). Among these, 15 patients (88%) also experienced seizures, with 10 developing eye–head movements prior to seizures and five afterward.

Episodic hypersomnolence was observed in 26 patients (29%), episodic psychiatric or behavioral abnormalities in 17 (19%), sleep disturbances in 13 (14%), migraines in eight (9%), periodic vomiting and attention-deficit hyperactivity disorder (ADHD)-like symptoms in seven (8%) each, strabismus in three (3%), and stroke-like episodes in three. Anxiety, depression, type II diabetes, and autism spectrum disorder were each noted in a single case.

Head circumference measurements were taken from 74 patients between 0.2 and 19 years of age (median: 4 years). Among them, 47% (35/74) presented with normal head circumferences, 26% (19/74) displayed microcephaly, and 27% (20/74) exhibited mildly reduced head circumferences.

### Familial cases

Eleven families with more than one case of Glut1DS, except a family with a pair of twins, were identified, accounting for 12.5% of the cohort. These families included 12 children and 12 adults, with one family comprising four members carrying *SLC2A1* variants. The inheritance pattern in these families was consistent with autosomal dominance, with *SLC2A1* variants inherited from the father in eight cases and from the mother in four cases. The identified variants included nine missense forms, one nonsense variant, and one frameshift mutation caused by a small insertion. Notably, three families carried the most prevalent variant, c.997C > T (p.R333W). Among the affected children, eight were male and four were female, with age of onset ranging from 3 to 120 months (median: 6 months). Of these cases, four presented with the early onset classic form, two with the late-onset classic form, and six with the non-classic form. The ages of the parents harboring *SLC2A1* variants ranged from 29 to 43 years (median: 37 years). Seven parents exhibited non-classic symptoms, including PED in four cases, migraines in two cases, and poor memory in one case. Five parents were asymptomatic, reflecting an estimated penetrance rate of 79%.

### Cerebrospinal fluid analysis

CSF glucose levels were measured in 73 patients (81%), ranging from 1.0 to 2.6 mmol/L (median: 1.9 mmol/L; IQR: 1.7–2.0 mmol/L). Among these, 66 patients (90%) exhibited CSF glucose levels below 2.2 mmol/L. CSF-to-blood glucose ratios were available for 71 patients (79%), ranging from 0.20 to 0.63 (median: 0.37; IQR: 0.33–0.42). Notably, 46 patients (65%) and 62 patients (87%) had ratios below 0.4 and 0.45, respectively. Interestingly, one patient with large fragment deletions, including an exon 1 deletion, and presenting with mild clinical symptoms such as predominantly paroxysmal movement disorder, had a CSF glucose level of 2.6 mmol/L and a CSF-to-blood glucose ratio of 0.63. The patient was consistent with the diagnosis of this disease. CSF lactate levels, measured in 40 patients (44%), ranged from 0.2 to 1.5 mmol/L (median: 0.9 mmol/L; IQR: 0.8–1.0 mmol/L). CSF cell counts were normal in all patients.

In patients with the early onset classic phenotype (*n* = 41), CSF glucose levels ranged from 1.0 to 2.2 mmol/L (median: 1.8 mmol/L; IQR: 1.5–1.9 mmol/L), with CSF-to-blood glucose ratios (*n* = 40) ranging from 0.22 to 0.53 (median: 0.35; IQR: 0.30–0.38). Lactate levels (*n* = 23) ranged from 0.2 to 1.5 mmol/L (median: 0.9 mmol/L; IQR: 0.8–1.1 mmol/L). Cases with the late-onset classic phenotype (*n* = 9) demonstrated CSF glucose levels ranging from 1.7 to 2.3 mmol/L (median: 2.1 mmol/L; IQR: 1.8–2.2 mmol/L) and ratios from 0.37 to 0.46 (median: 0.43; IQR: 0.41–0.44). Lactate levels in these cases (*n* = 5) ranged from 0.6 to 1.1 mmol/L (median: 0.9 mmol/L; IQR: 0.77–1.0 mmol/L). Patients with the non-classic phenotype (*n* = 23) exhibited CSF glucose levels between 1.6 and 2.6 mmol/L (median: 1.9 mmol/L; IQR: 1.8–2.1 mmol/L), with ratios from 0.28 to 0.63 (median: 0.39; IQR: 0.35–0.42). Lactate levels in this group (*n* = 12) ranged from 0.3 to 1.4 mmol/L (median: 0.9 mmol/L; IQR: 0.78–1.1 mmol/L). Significant differences in CSF glucose levels (*P* = 0.003) and CSF-to-blood glucose ratios (*P* = 0.001) were observed among the three groups (Table 1).

### Genetic characteristics

Sixty-nine unique heterozygous *SLC2A1* variants were identified among 90 patients, comprising 39 previously reported and 30 novel variants (Table S2). Of the unreported variants, 23 were classified as pathogenic, three as likely pathogenic, and four as variants of uncertain significance (VUS). All four VUS variants, c.412G > C (p.Gly138Arg), c.431 T > G (p.Val144Gly), c.875A > G (p.Tyr292Cys), and c.1243A > G (p.Asn415Asp), were located at highly conserved amino acid positions. Bioinformatics tools, such as MutationTaster, PolyPhen-2, SIFT, and CADD, predicted these variants to be deleterious. Structural modeling using pMOL software indicated that these amino acid substitutions disrupted protein stability by altering intramolecular interactions, confirming pathogenicity. Clinical presentations of these four patients, as well as CSF glucose levels below 2.2 mmol/L (1.97, 1.86, 2.08, and 1.88 mmol/L) and CSF-to-blood glucose ratios under 0.45 (0.42, 0.4, 0.43, and 0.38), satisfied the clinical diagnostic criteria for Glut1DS.

**Fig. 1:**
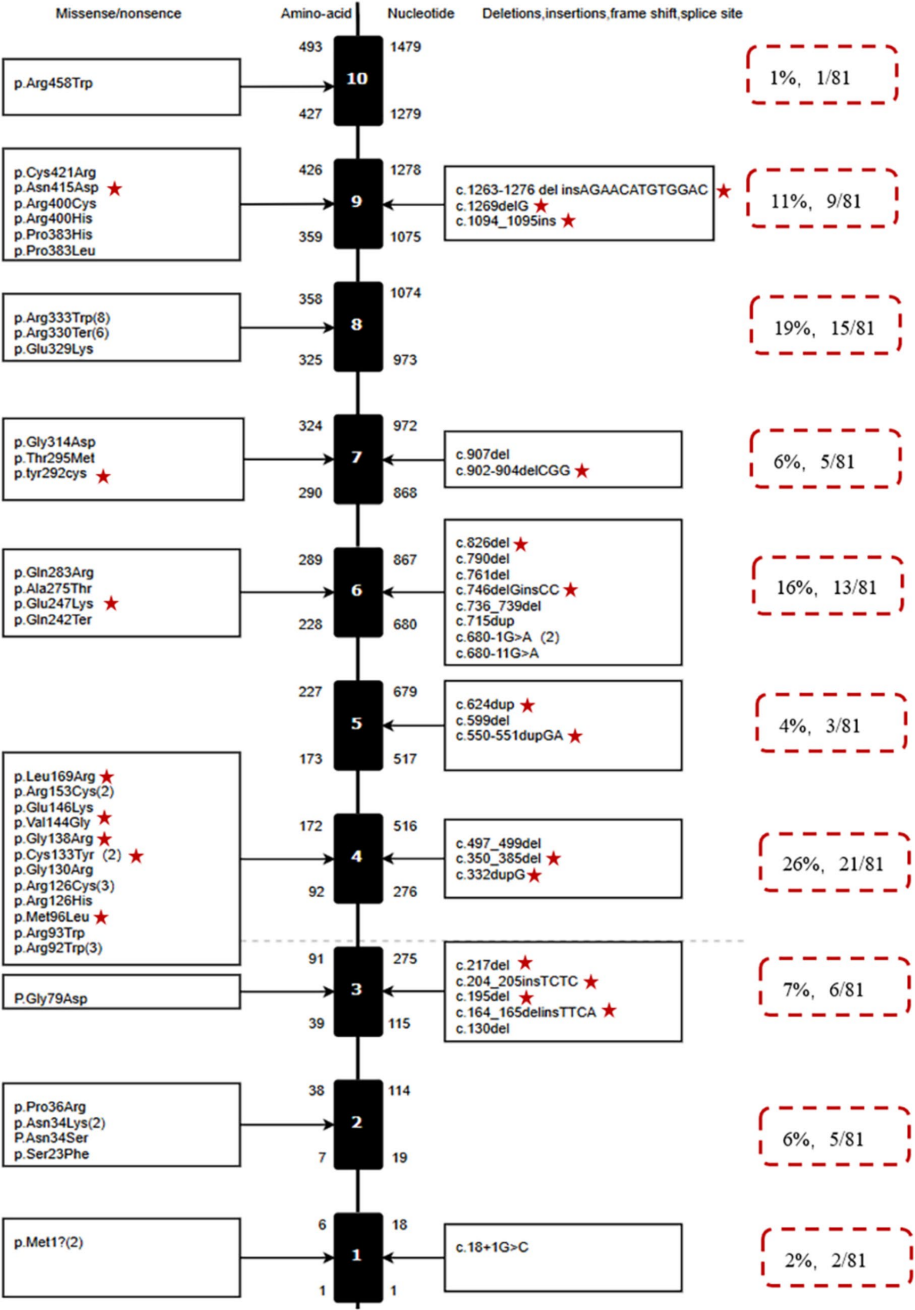
The mutation distribution map of the *SLC2A1* gene in Glut1DS patients is as follows: the black vertical line represents the *SLC2A1* gene, with exons denoted by boxes. Missense and nonsense mutations are displayed to the left of each box, while frameshift mutations, in-frame insertions/deletions, and splice-site mutations are shown to the right. Numbers in parentheses indicate the number of patients harboring each specific mutation. Cases of multi-exon/single-exon deletions or whole-gene deletions (not shown) include eight instances of novel mutations. Asterisks highlight mutation sites that have not been previously reported in the literature

Among the 69 variants, 46 (51%) were missense mutations, 19 (21%) were frameshift variants (10 deletions, six insertions, and three combined deletion–insertions), nine (10%) were single/multiple exon or whole-gene deletions, seven (8%) were nonsense mutations, four (4%) were splicing variants, three (3%) were in-frame deletions/insertions, and two (2%) affected start codons (Fig. 1). The most common variant, c.997C > T (p.Arg333Trp), was identified in eight probands (9%), followed by c.988C > T (p.Arg330*), c.274C > T (p.Arg92Trp), and c.376C > T (p.Arg126Cys), which were found in six, three, and three probands, respectively. The variants c.102 T > A (p.Asn34Lys), c.398G > A (p.Cys133Tyr), c.457C > T (p.Arg153Cys), c.2 T > A (p.Met1?), and c.680-1G > A were each found in two probands. The remaining 60 variants were unique to individual probands. There were no statistically significant differences in the distribution of *SLC2A1* gene variant types among the early onset classic, late-onset classic, and atypical groups (Table 1).

**Fig. 2:**
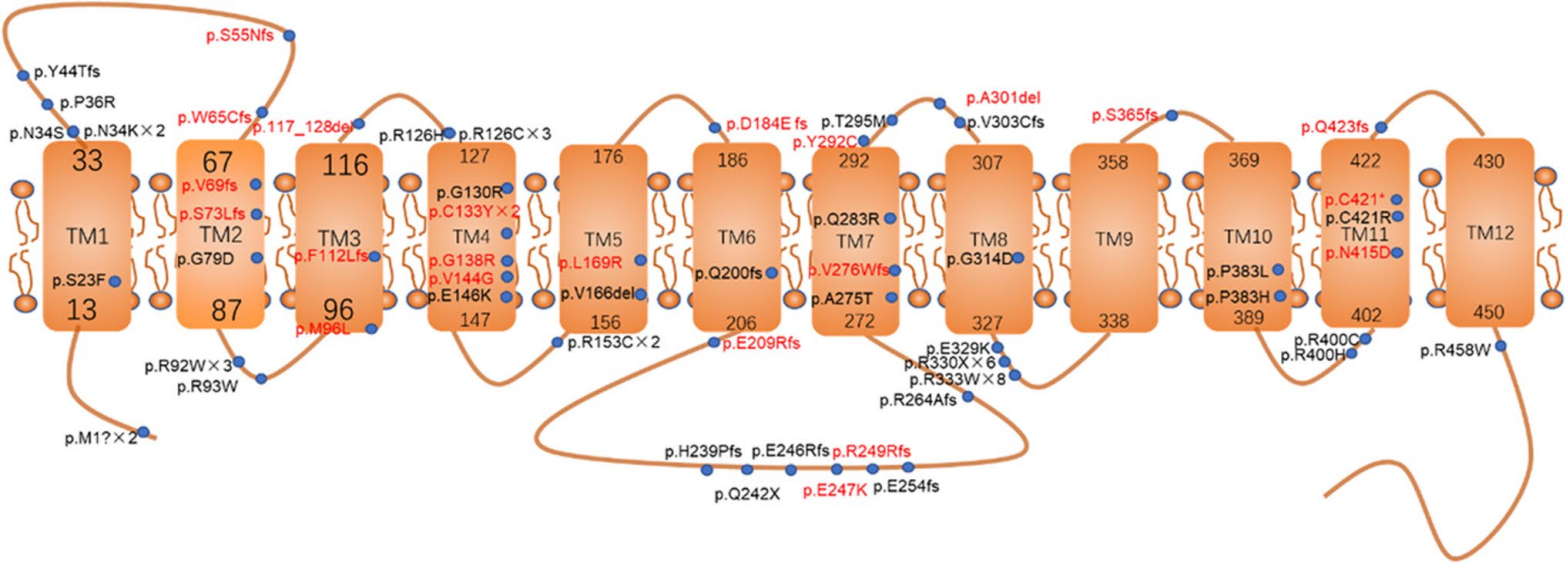
Distribution of *SLC2A1* gene mutation sites on the GLUT1 protein. The map illustrates 57 mutation sites identified in 77 patients with Glut1 deficiency syndrome (Glut1DS). Mutations involving multi-exon/single-exon deletions, whole-gene deletions (nine cases), and intron mutations (four cases) are not displayed. Mutation sites marked in red indicate novel mutations unreported previously. Among the mutations, 44% (34/77) are located in intracellular regions, 31% (24/77) in transmembrane regions, and 25% (19/77) in extracellular regions

Exon 4 was the most frequently affected, accounting for 26% of the variants, followed by exon 8, which accounted for 19%. The distribution of variants across the GLUT1 protein revealed that 44% (34/77) were in intracellular regions, 31% (24/77) were in transmembrane regions, and 25% (19/77) were in extracellular regions (Fig. 2).

### Treatment

#### Antiepileptic drug therapy

**Fig. 3:**
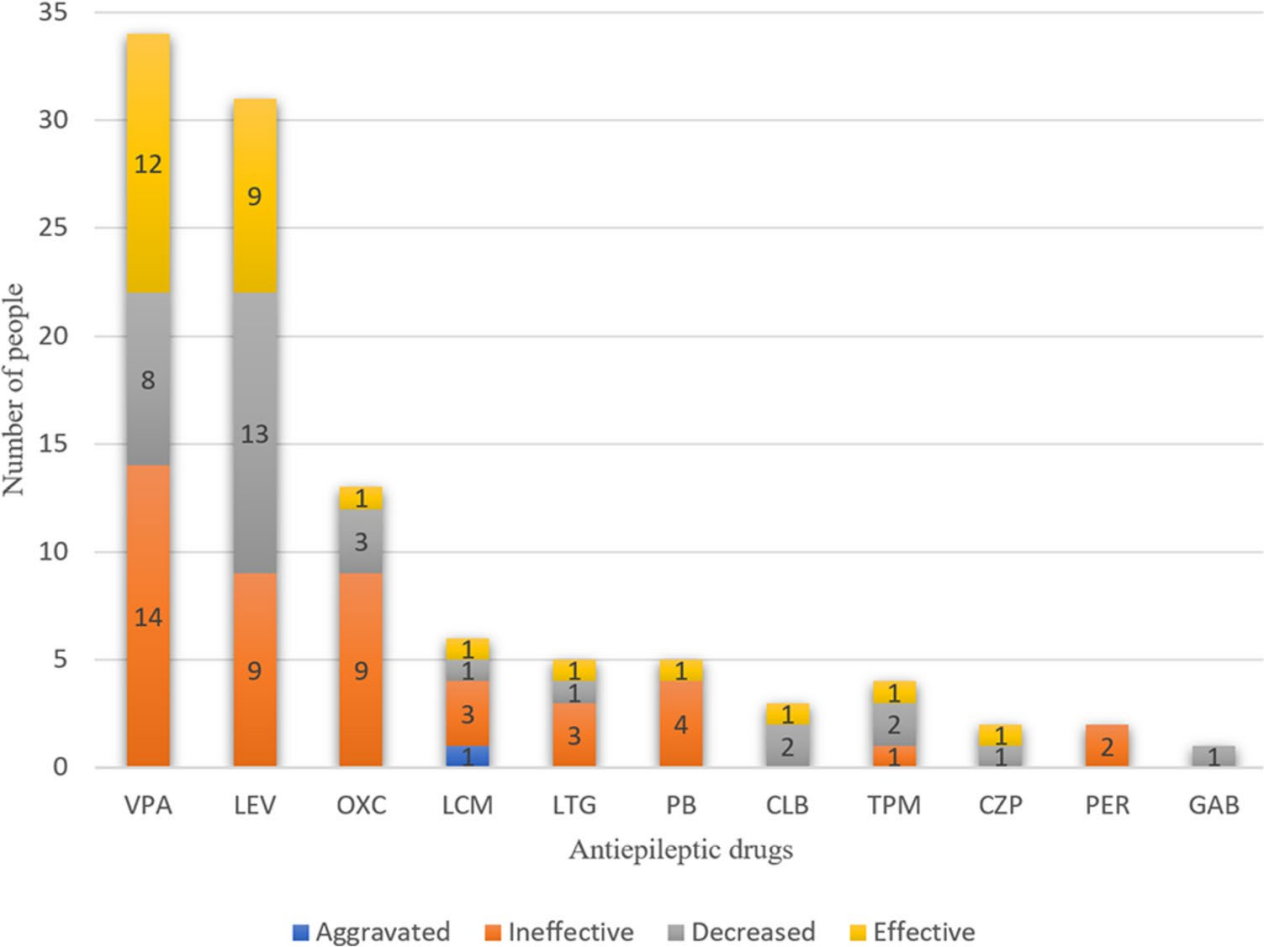
Efficacy of antiepileptic drugs (monotherapy/combination) in children with Glut1DS. *VPA* valproate, *LEV* levetiracetam, *OXC* oxcarbazepine, *LCM* lacosamide, *LTG* lamotrigine, *PB* phenobarbitone, *CLB* clobazam, *TPM* topiramate, *CZP* clonazepam, *PER* perampanel, *GAB* gabapentin

Among the 69 patients with seizures, 59 (87%) received antiepileptic drug (AED) therapy prior to initiating the ketogenic diet. Of these, 32 patients (53%) used one AED, 18 (30%) used two, six (10%) used three, and four (7%) utilized between four and seven AEDs. The most commonly used AEDs included valproate (34 cases) and levetiracetam (31 cases), followed by oxcarbazepine (13 cases), lacosamide (six cases), lamotrigine and phenobarbital (five cases each), topiramate (four cases), clobazam (three cases), clonazepam and perampanel (two cases each), and gabapentin (one case). Treatment efficacy details are summarized in Fig. 3. In general, AEDs showed some effects in controlling seizures, although the effect was limited.

#### Ketogenic diet

A total of 79 patients underwent ketogenic diet therapy, initiated at ages ranging from 0.25 to 14 years (median: 3.8 years). The duration of therapy varied between 0 and 14 years (median: 4.2 years). At the start of the ketogenic diet, 54 patients were receiving AED therapy concurrently. As of the last follow-up in October 2024, 73 patients remained on the diet, while six had discontinued it. AEDs had been successfully discontinued in 74% (40/54) of patients. Several types of ketogenic diets were used in our cohort, ranked by the number of patients as follows: classic ketogenic diet, medium-chain triglyceride (MCT) diet, and modified Atkins diet.

Among 57 patients with seizures, 47 (82%) achieved seizure freedom, including 40 on the ketogenic diet alone, five on the diet combined with AEDs, and two with an unknown regimen. Four patients experienced > 50% seizure reduction (one on the diet alone, three with AEDs), one experienced < 50% seizure reduction (with AEDs), and five showed no change in seizure frequency (all on a combined regimen). The timing of seizure resolution varied, with 28 patients achieving seizure freedom more than 1 month before ketogenic diet initiation, 15 within one week, one within two weeks, and three within 1 month. Among the five patients with partial seizure control, one improved within a week, three within 2 weeks, and one within a month of starting the diet. In addition to seizure control, 18 of the 47 patients with movement disorders experienced resolution of their symptoms, while 26 showed improvement, and three exhibited no changes. The improvement occurred between one week and six months after initiating the ketogenic diet. Among the 71 patients (including two who discontinued the diet) who adhered to the diet for more than six months, 48 demonstrated cognitive and language improvements, while 23 showed no significant changes.

Six patients discontinued the ketogenic diet after durations ranging from 0.3 to 72 months. Reasons for discontinuation included poor compliance (three patients), severe metabolic acidosis with associated diarrhea and refusal to eat (one ), lack of efficacy accompanied by abdominal pain, vomiting, and increased seizure frequency (one ), and lack of efficacy with increased seizure frequency (one ).

Adverse reactions at the initiation phase of the ketogenic diet were reported in 23 patients, who experienced one to four side effects each. The most common initial side effects were diarrhea or vomiting, followed by metabolic acidosis, hypoglycemia, constipation, electrolyte disturbances, abdominal pain, and liver dysfunction. All adverse events were manageable. During the maintenance phase, 32 patients reported side effects, with the most common being elevated uric acid levels, followed by hyperlipidemia, constipation, hair loss, weight loss or stunted growth, urinary tract stones, intermittent diarrhea, and intermittent abdominal pain. Despite these side effects, the ketogenic diet was well tolerated overall, with most adverse reactions being transient and treatable.

## Discussion

Glut1DS is a treatable neurogenetic metabolic disorder, with the majority of cases being sporadic. Approximately 90% of cases are caused by de novo heterozygous mutations in the *SLC2A1* gene, following an autosomal dominant inheritance pattern, while a minority exhibit autosomal recessive inheritance [9]. Most familial cases demonstrate autosomal dominant inheritance with incomplete penetrance. The specific inheritance pattern depends on the relative pathogenicity of *SLC2A1* mutations and the degree of haploinsufficiency. In our cohort, familial cases accounted for 12.5% with autosomal dominant inheritance. Missense mutations were the predominant type, with most *SLC2A1* mutations inherited paternally. Probands generally exhibited more severe symptoms with earlier onset compared to their parents, who are often mildly symptomatic or asymptomatic carriers. This is understandable, as severely affected individuals tend not to have children, whereas asymptomatic patients may pass on the condition.

Glut1DS manifests in two major clinical phenotypes: classic and non-classic. The classic type constitutes approximately 90% of cases, with early onset (within 1 to 6 months after birth) and late-onset (> 2 years) subcategories [10]. The non-classic phenotype, accounting for approximately 10% of cases, typically has later onset and a milder presentation, often including PED with or without seizures, paroxysmal or persistent movement disorders with episodic worsening, and developmental delays. In our cohort, classic cases accounted for 70%, while non-classic cases accounted for 30%, underscoring how advancements in next-generation sequencing have expanded the spectrum of clinical phenotypes by identifying atypical cases.

The clinical features of Glut1DS are age-specific, with episodic eye–head movements and seizures often presenting in infancy, followed by developmental delays, movement disorders, and ataxia emerging later. In adolescent and adult patients, movement disorders are the most frequently reported symptoms [8]. Leary et al. [11] reported that generalized tonic–clonic and absence seizures were the most frequent types in the cohort. Early onset absence epilepsy (before four years of age), myoclonic–atonic epilepsy, idiopathic generalized epilepsy, childhood absence epilepsy, and eyelid myoclonia epilepsy [12] could be associated with pathogenic *SLC2A1* mutations. Additionally, patients with Glut1DS can experience epileptic spasms. Among our cohort, 77% (69/90) of patients experienced seizures, with 36% (25/69) exhibiting two or more seizure types. Generalized seizures (64%, 44/69) were more prevalent than focal seizures (48%, 33/69). Generalized tonic–clonic and myoclonic seizures predominated, followed by absence, atonic, generalized tonic, myoclonic–atonic seizures, and eyelid myoclonia. No spasms were observed in our cohort.

In pediatric patients, symptoms such as abnormal eye and head movements may precede seizure onset, typically within six months after birth. The eye movements are rapid, brief, multidirectional, and occur at intervals of 200–800 ms, often accompanied by frequent head movements in the same direction [13]. This unique paroxysmal eye–head movement, associated with the deficiency in the high energy demands of the developing visual system, represents a hallmark symptom in the early stages of Glut1DS, second only to seizures. Pearson et al. [13] found that 15 out of 18 cases presented with paroxysmal eye–head movements within the first six months of life, and seven out of eight continued to experience them until age six. Ten of 16 children exhibited these movements prior to the onset of seizures. The incidence of paroxysmal eye head movement in our cohort was 19%, which is lower than reported in the literature. This may be related to parental neglect during early stages of the disease. Clinicians should focus on the presence of this symptom when taking the medical history of a child with suspected symptoms.

Diagnostic markers of Glut1DS include diminished CSF glucose levels (< 2.8 mmol/L), with more than 90% of patients having levels < 2.2 mmol/L, a reduced CSF-to-blood glucose ratio (< 0.45 in most cases), and normal or slightly low lactate levels [8, 14]. Differential diagnoses include other conditions causing low CSF glucose, such as hypoglycemia, central nervous system (CNS) infections, genetic metabolic disorders, subarachnoid hemorrhage, and status epilepticus. Notably, disorders such as neurodevelopmental disorders with visual defects and brain anomalies caused by monoallelic HK1 variants should also be differentiated. Although HK1 variants may exhibit low CSF glucose levels similar to Glut1DS, HK1-associated NDD [15] are characterized by elevated CSF lactate levels. In our cohort, 90% (66/73) of patients exhibited low CSF glucose levels below 2.2 mmol/L, and 87% (62/73) showed a CSF-to-blood glucose ratio below 0.45, highlighting its critical role in diagnosis.

Glut1DS is primarily caused by pathogenic mutations in *SLC2A1*, with about 90% of patients harboring the mutations. Among these, 90% arise de novo, while 10% are inherited. The mutations include missense, deletions, insertions, splice-site, and nonsense variants (84%), with 13% consisting of single- or multi-exon deletions or complete gene deletions [10, 16]. Less common mutations, such as intronic and start codon variants, have also been reported. For patients with a strong clinical suspicion but negative results in exonic sequencing and copy-number variation testing, MLPA and non-coding region analyses should be considered [17]. Since the initial discovery of *SLC2A1* mutations as the cause of Glut1DS by Seidner et al. [18] in 1998, more than 430 mutations have been identified. Mutation hotspots include Asn34, Gly91, Ser113, Arg126, Arg153, Arg264, Thr295, Arg330, and Arg333 [10, 19–21]. In our cohort, the most frequently observed mutations were Arg333 (eight cases), Arg330 (six cases), Asn34 and Arg126 (three cases each), Arg153 (two cases), and Arg264 and Thr295 (one case each). Notably, high-frequency mutations specific to Chinese patients included Arg92 (three cases), Cys133 (two cases), and Met1 (two cases). The remaining 60 variants were unique to individual probands. Exon 4 represented a significant mutation hotspot, accounting for 24% of mutations globally [22] and 27% in our cohort. Additionally, 10% of patients in our study exhibited substantial fragment deletions.

The ketogenic diet is the first-line treatment for Glut1DS. A study in 2020 by Schwantje et al. [23] involving 270 Glut1DS patients, highlighted its efficacy: 83% of epileptic patients showed improvement, while 17% remained unchanged. Among 127 patients with movement disorders, 82% improved, 17% remained stable, and 1% worsened. For 58 patients with cognitive impairments, 59% improved, 40% remained stable, and 1% worsened. Seizures responded within days to weeks of ketogenic therapy initiation, with earlier treatment yielding better outcomes. Movement and cognitive impairments improved within months. Notably, these patients followed the classic ketogenic diet and the Modified Atkins diet. It appears that the efficacy of the classic ketogenic diet in controlling seizures is higher than that of the modified Atkins diet. In our cohort, 82% of patients achieved complete resolution of seizures following ketogenic diet therapy, while 7% experienced a seizure reduction of over 50%, 2% had a reduction of less than 50%, and 9% showed no improvement. Among 49 patients with movement disorders, 38% experienced complete resolution, 55% showed improvement, and 7% exhibited no change. The time required for symptom improvement ranged from 1 week to 6 months. Among 71 patients who adhered to the diet for over 6 months, 68% demonstrated cognitive and language improvements, while 32% showed no significant change. Cognitive improvements in our cohort exceeded previous findings, underscoring the need for stricter developmental scales in future evaluations.

For patients unresponsive to ketogenic therapy [24], alternative approaches have been or will be explored, including gene replacement therapy, GLUT1 protein supplementation [25], targeted compounds [26], zinc sulfate, sodium lactate [27], diazoxide [28], acetazolamide [29], and exchange transfusion [30]. However, these treatments remain experimental, and further validation is required.

In vitro studies [8] reveal that certain compounds, such as caffeine, phenobarbital, diazepam, valproate, ethanol, and chloral hydrate, inhibit glucose transport, whereas carbamazepine, phenytoin, topiramate, and zonisamide do not exhibit such effects. Hence, anticonvulsants lacking inhibitory properties should be preferred for Glut1DS patients with suboptimal responses to ketogenic therapy. Although valproate reduces GLUT1 activity in red blood cells by 20% to 30% in vitro, it was effective in controlling seizures in some patients in our cohort, highlighting discrepancies between in vitro and clinical outcomes [31]. Thus, valproate should be used cautiously, but should not be categorically contraindicated in Glut1DS management.

In conclusion, this study describes the phenotypic and genotypic characteristics of 90 patients with Glut1DS, expanding the clinical and genetic spectrum of the disorder. The ketogenic diet remains the first-line treatment and is generally well tolerated by patients. Early identification and diagnosis followed by the prompt initiation of a ketogenic diet can significantly improve prognosis. However, some children do not respond to the ketogenic diet, highlighting the need for further research into alternative therapies.

## Supplementary Information

Below is the link to the electronic supplementary material.

Supplementary file1 (DOCX 63 KB)

## Data Availability

The datasets used and/or analyzed during the current study are available from the corresponding author on reasonable request.
