# Supplementary material for: Clinical and genetic characteristics of glucose transporter 1 deficiency syndrome in a large cohort of Chinese patients
Source: World J Pediatr. 2025 Mar 6;21(3):274–83. doi: 10.1007/s12519-025-00884-9 (PMC11958367; doi:10.1007/s12519-025-00884-9)
Supplement: Supplementary file 1 — Supplementary file1 (DOCX 63 KB) [file 12519_2025_884_MOESM1_ESM.docx]

**Supplementary table S1** Clinical features of 90 Glut1DS.

**S1.1** Clinical features of 53 patients with classical phenotype.

| Characteristics of patients | | | | | Clinical data | | | | Biochemical data | | | | Effect of ketogenic diet on | | |
| --- | --- | --- | --- | --- | --- | --- | --- | --- | --- | --- | --- | --- | --- | --- | --- |
| Patient | Sex | Age at diagnosis (m) | Age at last review (Y) | Age at seizure | | dyskinesia | Mental retardation | microcephaly | | CSF glucose (mmol/L) | CSF: blood glucose ratio | CSF  Lactate (mmol/L) | Seizures | Movement  disorder | Cognition |
| 1 | M | 6 | 5.6 | 4.0 | | N | Mild | Y | | 1.7 | 0.30 | 1.2 | D | N/A | I |
| 2 | F | 60 | 8.9 | 2.0 | | P | Ver | N | | 1.9 | 0.34 | 0.8 | D | D | I |
| 3 | F | 60 | 8.9 | 2.0 | | P | Ver | N | | 1.8 | 0.35 | 0.8 | D | R | I |
| 4 | M | 142 | 14.1 | 1.5 | | C/P | Mild | N | | 1.9 | 0.30 | 0.6 | D | N | N |
| 5 | M | 4 | 14.3 | 1.4 | | N | Norm | Y | | 1.2 | 0.23 | 0.2 | D | N/A | I |
| 6 | M | 70 | 13.7 | 22.0 | | P | Mild | N | | 1.5 | 0.53 | NA | D | D | I |
| 7 | F | 23 | 5.8 | 3.0 | | N | Mod | Y | | 1.8 | 0.39 | NA | D | N/A | N |
| 8 | F | 32 | 4.7 | 18.0 | | C | Mild | NA | | NA | NA | NA | D | R | I |
| 9 | F | 7 | 0.6 | 2.0 | | N | Sev | S | | 1.7 | 0.35 | NA | N | N/A | N/A |
| 10 | M | 38 | 10.0 | 1.5 | | C/P | Mild* | NA | | 1.7 | 0.35 | 0.9 | R | R | I |
| 11 | M | 21 | 10.7 | 11.0 | | P | Mild | Y | | 1.9 | 0.46 | 1.2 | N | D | N |
| 12 | M | 32 | 10.9 | 2.0 | | N | Mod* | Y | | 1.1 | 0.36 | NA | D | D | I |
| 13 | F | 30 | 7.7 | 21.0 | | C | Mod* | S | | 1.9 | 0.33 | 0.9 | D | D | I+ |
| 14 | F | 10 | 5.0 | 8.0 | | C | Mod | S | | 1.0 | 0.22 | NA | D | R | I |
| 15 | M | 4 | 13.7 | 2.0 | | N | Mild* | N | | 1.2 | 0.25 | NA | D | N/A | I |
| 16 | M | 74 | 19.0 | 1.1 | | C/P | Ver | N | | 1.9 | 0.37 | 0.9 | / | / | / |
| 17 | M | 51 | 5.4 | 15.0 | | C | Mild | Y | | 1.9 | 0.38 | 1.5 | D | R | I |
| 18 | F | 55 | 16.0 | 15.0 | | P | Norm | Y | | 2.1 | 0.38 | NA | / | / | / |
| 19 | M | 28 | 11.7 | 4.0 | | N | Ver* | NA | | NA | NA | NA | D | N/A | I |
| 20 | F | 9 | 11.4 | 4.0 | | N | Ver | Y | | 2.1 | 0.37 | NA | N | N/A | N/A |
| 21 | F | 41 | 7.6 | 5.0 | | C/P | Mild | NA | | NA | NA | NA | D | D | I |
| 22 | F | 125 | 20.8 | 10.0 | | P | Mild* | N | | 1.8 | 0.36 | 0.7 | / | / | / |
| 23 | M | 80 | 8.9 | 5.0 | | N | Sev | S | | NA | NA | NA | N | N/A | N |
| 24 | M | 16 | 3.0 | 10.0 | | N | Norm | S | | 2.0 | 0.42 | 0.9 | D | N/A | I |
| 25 | M | 27 | 7.9 | 3.0 | | N | Ver | Y | | 1.6 | 0.32 | 0.9 | D | R | I |
| 26 | F | 12 | 7.1 | 6.0 | | C | Mild | S | | 1.7 | 0.31 | NA | D | R | I |
| 27 | M | 7 | 5.6 | 1.3 | | C | Mod | NA | | 1.9 | 0.37 | NA | D | R | I |
| 28 | F | 60 | 13.4 | 3.0 | | C/P | Mild* | N | | 1.8 | 0.33 | 1.4 | N | N | N |
| 29 | F | 20 | 7.7 | 4.0 | | C/P | Mild | Y | | NA | NA | NA | D | R | I |
| 30 | M | 27 | 10.8 | 6.0 | | C/P | Ver | Y | | 1.6 | 0.28 | NA | D | R | I |
| 31 | F | 27 | 3.6 | 16.0 | | N | Norm | NA | | NA | NA | NA | D | N/A | I |
| 32 | M | 20 | 4.2 | 17.0 | | C | Mild | S | | 2.2 | 0.48 | 1.0 | D | R | I |
| 33 | M | 52 | 11.4 | 10.0 | | C/P | Mod* | NA | | NA | NA | NA | D | R | I |
| 34 | M | 84 | 9.0 | 8.0 | | N | Mod | S | | NA | NA | NA | D | N/A | N |
| 35 | M | 63 | 8.1 | 3.0 | | N | Ver | S | | 2.1 | 0.38 | 1.0 | D | N/A | I |
| 36 | F | 2.4 | 4.2 | 2.4 | | N | Norm* | N | | 1.1 | 0.20 | NA | D | N/A | I |
| 37 | M | 73 | 15.6 | 9.0 | | N | Ver* | Y | | 1.9 | 0.37 | NA | / | / | / |
| 38 | F | 43 | 7.7 | 18.0 | | N | Mild* | S | | 1.5 | 0.29 | 0.8 | D | N/A | I |
| 39 | M | 5 | 6.3 | 1.3 | | N | Ver* | NA | | 2.2 | 0.49 | NA | D | N/A | I |
| 40 | M | 22 | 7.2 | 2.0 | | C | Mod | S | | 2.1 | 0.51 | 1.1 | D | D | I |
| 41 | M | 84 | 13.2 | 6.0 | | P | Ver | Y | | 1.7 | NA | 1.0 | D | D | N |
| 42 | M | 2 | 11.3 | 1.8 | | C | Mod* | S | | 1.9 | 0.43 | NA | / | / | / |
| 43 | M | 77 | 15.7 | 12.0 | | C/P | Ver | N | | 1.5 | 0.33 | 0.7 | D | D | I |
| 44 | M | 110 | 13.2 | 22.0 | | C | Mod | N | | 2.0 | 0.36 | NA | D | R | N |
| 45 | M | 21 | 6.9 | 5.0 | | N | Mild | N | | 1.4 | 0.27 | 0.7 | D | R | N |
| 46 | F | 40 | 10.0 | 1.5 | | C/P | Mild* | NA | | NA | NA | NA | R | N/A | I |
| 47 | F | 5 | 6.4 | 2.0 | | N | Mild* | NA | | 1.4 | 0.29 | NA | D | N/A | I |
| 48 | M | 5 | 2.0 | 3.3 | | N | Mild* | S | | NA | NA | NA | / | / | / |
| 49 | M | 51 | 8.4 | 2.0 | | C | Ver* | N | | NA | NA | NA | D | R | I |
| 50 | M | 48 | 6.2 | 3.0 | | C/P | Ver* | NA | | 1.7 | 0.35 | NA | D | R | N |
| 51 | M | 30 | 3.1 | 3 | | C | Mild | N | | 1.45 | 0.29 | 1.1 | D | N/A | N/A |
| 52 | M | 4 | 0.8 | 3 | | N | Mild* | N | | 1.55 | 0.26 | 0.59 | D | N/A | N/A |
| 53 | F | 3 | 0.7 | 1 | | N | Nrom | N | | 1.1 | 0.21 | 0.8 | D | N/A | N/A |

**S1.2** Clinical features of 10 patients with late-classical phenotype.

| Characteristics of patients | | | | | | Clinical data | | | | Biochemical data | | | Effect of ketogenic diet on | | |
| --- | --- | --- | --- | --- | --- | --- | --- | --- | --- | --- | --- | --- | --- | --- | --- |
| Patient | Sex | Age at diagnosis(m) | Age at last review (Y) | Age at seizure | dyskinesia | | Mental retardation | microcephaly | CSF glucose (mmol/L) | | CSF: blood glucose ratio | CSF  Lactate (mmol/L) | Seizures | Movement  disorder | Cognition |
| 54 | F | 110 | 12.4 | 88.0 | N | | Ver * | N | 2.3 | | 0.44 | 0.6 | D | N/A | N |
| 55 | M | 62 | 9.3 | 48.0 | N | | Ver | NA | 2.1 | | 0.43 | NA | D | N/A | I |
| 56 | M | 51 | 9.6 | 30.0 | C/P | | Ver | S | 1.8 | | 0.37 | NA | D | D | I |
| 57 | M | 175 | 22.2 | 144.0 | N | | Mild | N | 2.2 | | 0.46 | NA | D | N/A | N/A |
| 58 | F | 64 | 7.7 | 41.0 | N | | Mild* | NA | 1.7 | | 0.41 | 0.9 | R | N/A | I |
| 59 | M | 34 | 6.1 | 33.0 | P | | Mild | N | 2.1 | | 0.44 | 1.0 | N | R | N |
| 60 | F | 44 | 10.7 | 33.0 | C/P | | Mild | S | 1.8 | | 0.41 | NA | D | R | I |
| 61 | F | 108 | 12.4 | 6.0 | C/P | | Mild | N | 2.2 | | 0.45 | 1.1 | R | R | N |
| 62 | F | 48 | 7.4 | 43.0 | C | | Mild | S | NA | | NA | NA | D | R | I |
| 63 | M | 125 | 10.5 | 27.00 | N | | Mild | N | 2.0 | | 0.38 | 0.77 | D | N/A | N/A |

**S1.3** Clinical features of 27 patients with non-classical phenotype.

| Characteristics of patients | | | | | Clinical data | | | | | Biochemical data | | | | | Effect of ketogenic diet on | | | | | |  |
| --- | --- | --- | --- | --- | --- | --- | --- | --- | --- | --- | --- | --- | --- | --- | --- | --- | --- | --- | --- | --- | --- |
| Patient | Sex | Age at diagnosis (m) | Age at last review (Y) | Age at seizure(m) | | dyskinesia | Mental retardation | microcephaly | CSF glucose (mmol/L) | | CSF: blood glucose ratio | | CSF  Lactate (mmol/L) | | Seizures | | Movement  disorder | | Cognition | |  |
| 64 | M | 128 | 22.3 | N | | P | Norm | N | 1.9 | | | 0.41 | | 0.4 | | N/A | | D | | N | |
| 65 | F | 228 | 27.6 | N | | P | Ver* | N | NA | | | NA | | NA | | / | | / | | / | |
| 66 | F | 69 | 19.4 | 66(1 time) | | P | Mild | S | 1.9 | | | 0.38 | | NA | | N/A | | N/A | | N/A | |
| 67 | F | 89 | 11.6 | 48(1 time) | | P | Norm | S | 1.9 | | | 0.40 | | NA | | N/A | | D | | N | |
| 68 | M | 57 | 17.2 | 24(1 time) | | C/P | Mild | N | 1.8 | | | 0.35 | | NA | | N/A | | D | | I | |
| 69 | M | 44 | 5.1 | N | | P | Norm | N | 2.6 | | | 0.63 | | 1.4 | | N/A | | R | | N | |
| 70 | F | 84 | 10.0 | N | | C/P | Mild | N | 2.1 | | | 0.42 | | 1.0 | | N/A | | R | | N | |
| 71 | M | 79 | 22.4 | N | | N | Ver | N | 2.3 | | | 0.50 | | NA | | / | | / | | / | |
| 72 | F | 75 | 14.8 | 56(1 time) | | C | Mild | N | 2.0 | | | 0.44 | | 1.0 | | N/A | | R | | N | |
| 73 | F | 27 | 4.2 | N | | P | Ver* | Y | 1.9 | | | 0.41 | | NA | | N/A | | D | | I | |
| 74 | F | 62 | 8.1 | N | | P | Mild | N | 1.7 | | | 0.28 | | 0.8 | | N/A | | R | | I | |
| 75 | M | 21 | 7.9 | N | | C/P | Mild* | Y | 1.9 | | | 0.35 | | 0.8 | | / | | / | | / | |
| 76 | M | 51 | 9.4 | N | | C/P | Mild | N | 1.6 | | | 0.33 | | NA | | N/A | | D | | I | |
| 77 | F | 22 | 9.9 | N | | C | Mild* | Y | 1.7 | | | 0.33 | | NA | | N/A | | R | | I | |
| 78 | F | 22 | 4.9 | N | | C | Mod | S | 1.8 | | | 0.34 | | NA | | N/A | | N/A | | N | |
| 79 | F | 38 | 12.3 | N | | P | Ver | Y | 2.2 | | | 0.34 | | 1.4 | | N/A | | D | | I | |
| 80 | M | 63 | 16.8 | N | | P | Ver | N | 1.9 | | | 0.41 | | 0.7 | | N/A | | N/A | | N/A | |
| 81 | F | 54 | 10.9 | N | | P | Ver* | NA | 2.3 | | | 0.40 | | NA | | N/A | | D | | I | |
| 82 | F | 21 | 3.1 | N | | C | Ver | Y | 1.9 | | | NA | | NA | | N/A | | R | | I | |
| 83 | M | 48 | 10.5 | N | | C/P | Ver* | NA | NA | | | NA | | N A | | N/A | | R | | N | |
| 84 | F | 47 | 5.2 | N | | P | Ver | N | 1.9 | | | 0.37 | | 1.2 | | N/A | | D | | N | |
| 85 | M | 24 | 3.6 | N | | N | Mild | S | NA | | | NA | | NA | | N/A | | N/A | | I | |
| 86 | M | 29 | 7.3 | 20/27(2 times) | | P | Mild | N | 1.9 | | | 0.43 | | 0.8 | | N/A | | R | | I | |
| 87 | F | 91 | 22.0 | N | | C/P | Norm | N | 1.7 | | | 0.37 | | NA | | / | | / | | / | |
| 88 | M | 51 | 10.5 | 24/36(2 times) | | C/P | Mod | N | 2.2 | | | 0.43 | | 0.3 | | N/A | | D | | I | |
| 89 | F | 78 | 10.4 | N | | C/P | Ver | Y | 1.8 | | | 0.36 | | 1.0 | | N/A | | D | | N | |
| 90 | F | 14 | 1.5 | N | | N | Mild* | NA | NA | | | NA | | NA | | / | | / | | / | |

Abbreviations: Sex: M, male; F, female; Movement disorder: C, continue; P, paroxysmal; C/P, continue with paroxysmal worsening; Mental retardation: verge (ver), IQ 70-85; mild, IQ 50–69; moderate (mod), IQ 30–49; severe-very severe (sev), (IQ <30; *, IQ test was not performed, the severity of mental retardation was based on school level and/or clinical impression of the treating physician; Microcephaly: Y, more than two standard deviations below the mean; S, within two standard deviations from the mean; Effect of ketogenic diet on (i) seizures: D, seizure disappeared; R, reduction of seizures; N, no reduction of seizures; /, Unketogenic Diet Therapy; (ii) movement disorder: D, disappearance of movement disorder; R, reduction of frequency and/or severity of movement disorder; N, no effect on movement disorder; (iii) cognition: I, subjective improvement; N, no change; N, No; NA, data not available; N/A, not applicable.

**Table S2** *SLC2A1* gene vaiants and classifications of genotype and phenotype.

| Exon | Nucleotide | Amino  acid | Location | Numbers of cases | Phenotype |
| --- | --- | --- | --- | --- | --- |
| 1 | c.2T>C | p.Met1? | Transcription codon | 64 | C |
| 1 | c.2T>A | p.Met1? | Transcription codon | 35 | A |
| Intron1 | c.18+1G>C | - | - | 4 | A |
| 2 | c.68C>T | p.Ser23Phe | Helix 1 | 70 | C |
| 2 | c.101A>G | P.Asn34Ser | Cytoplasmic loop 1–2 | 13 | A |
| 2 | c.102T>A | p.Asn34Lys | Cytoplasmic loop 1–2 | 2,3 | A |
| 2 | c.107C>G | p. Pro36Arg | Cytoplasmic loop 1–2 | 90 | C |
| 3 | c.130delT | p.Tyr44ThrfsTer34 | Cytoplasmic loop 1–2 | 1 | A |
| 3 | c.164_165delinsTTCA | P.Ser55AsnfsTer24 | Cytoplasmic loop 1–2 | 47 | A |
| 3 | c.195 delG | p.Trp65Cysfs*13 | Cytoplasmic loop 1–2 | 36 | A |
| 3 | c.204_205insTCTC | p.V69fs | Helix 2 | 51 | A |
| 3 | c.217delT | p.Ser73Leu fs*5 | Helix 2 | 38 | A |
| 3 | c.236G>A | P.Gly79Asp | Helix 2 | 14 | A |
| 4 | c.274C>T | p.Arg92Trp | Cytoplasmic loop 2–3 | 74 | C |
| 4 | c.274C>T | p.Arg92Trp | Cytoplasmic loop 2–3 | 8 | B |
| 4 | c.274C>T | p.Arg92Trp | Cytoplasmic loop 2–3 | 31 | A |
| 4 | c.277C>T | p.Arg93Trp | Cytoplasmic loop 2–3 | 72 | C |
| 4 | c.286A>C | p.Met96Leu | Helix 3 | 63 | B |
| 4 | c.332dupG | p.Phe112Leufs*9 | Helix 3 | 19 | A |
| 4 | c.350_385del | p.117_128del | Cytoplasmic loop 3–4 | 37 | A |
| 4 | c.376C>T | P.Arg 126 Cys | Cytoplasmic loop 3–4 | 25,49 | A |
| 4 | c.376C>T | p.Arg126Cys | Cytoplasmic loop 3–4 | 84 | C |
| 4 | c..377G>A | p.Arg126His | Helix 4 | 44 | A |
| 4 | c.388G>C | p.Gly130Arg | Helix 4 | 85 | C |
| 4 | c.398G＞A | P.Cys133Tyr | Helix 4 | 22 | A |
| 4 | c.398G＞A | P.Cys133Tyr | Helix 4 | 59 | B |
| 4 | c.412G>C | p.Gly138Arg | Helix 4 | 24 | A |
| 4 | c.431 T>G | p.Val144Gly | Helix 4 | 67 | C |
| 4 | c.436G>A | p.Glu146Lys | Helix 4 | 77 | C |
| 4 | c.457C>T | p.Arg153Cys | Cytoplasmic loop 4–5 | 60 | B |
| 4 | c.457C>T | P.Arg 153 Cys | Cytoplasmic loop 4–5 | 23 | A |
| 4 | c.497_499del | p.Val166del | Helix 5 | 73 | C |
| 4 | c.506T>G | p.Leu169Arg | Helix 5 | 46 | A |
| 5 | c.550-551 dup GA | p.Asp184Glu fs*8 | Cytoplasmic loop 5–6 | 88 | C |
| 5 | c.599delA | p.Gln200fs*29 | Cytoplasmic loop 6–7 | 16 | A |
| 5 | c.624 dupC | p.Glu209Argfs*28 | Cytoplasmic loop 6–7 | 41 | A |
| Intron 5 | c.680-11G>A | - | - | 80 | C |
| Intron 5 | c.680-1G>A | - | - | 28,52 | A |
| 6 | c.715dup | p.His239ProfsTer2 | Cytoplasmic loop 6–7 | 81 | C |
| 6 | c.724C>T | p.Q242Ter | Cytoplasmic loop 6–7 | 53 | B |
| 6 | c.736_739del | p.Glu246ArgfsTer5 | Helix 7 | 82 | C |
| 5 | C.741 G>A | P.Glu247Lys | Cytoplasmic loop 6–7 | 15 | A |
| 6 | c.746delGinsCC | p.Arg249Argfs*132 | Cytoplasmic loop 6–7 | 57 | B |
| 6 | C.761delA | p.Glu254fs | Cytoplasmic loop 6–7 | 87 | C |
| 6 | c.790del | p.Arg264Alafs*76 | Helix 7 | 32 | A |
| 6 | c.823G>A | p.Ala275Thr | Helix 7 | 54 | B |
| 6 | c.826delG | p.Val276Trpfs*64 | Helix 7 | 39 | A |
| 6 | c.848A>G | p.Gln283Arg | Helix 7 | 34 | A |
| 7 | c.875A>G | p.Tyr292cys | Helix 7 | 55 | B |
| 7 | c.884C>T | p.Thr295Met | Cytoplasmic loop 7–8 | 61 | B |
| 7 | c.902-904del CGG | p.Arg301del | Helix 8 | 26 | A |
| 7 | c.907delG | p.Val303Cysfs*37 | Cytoplasmic loop 7–8 | 27 | A |
| 7 | c.941G>A | p.Gly314Asp | Helix 8 | 58 | B |
| 8 | c.985G>A | p.Glu329Lys | Cytoplasmic loop 8–9 | 68 | C |
| 8 | c.988C>T | p.Arg330X | Cytoplasmic loop 8–9 | 10,40,43 | A |
| 8 | c.988C>T | p.Arg330X | Cytoplasmic loop 8–9 | 75,79,83 | C |
| 8 | c.997C>T | p.Arg333Trp | Cytoplasmic loop 8–9 | 65,76 | C |
| 8 | c.997C>T | p.Arg333Trp | Cytoplasmic loop 8–9 | 6,18,21,29,48,50 | A |
| 9 | c.1094_1095ins | p. Ser365fs | Cytoplasmic loop 9–10 | 33 | A |
| 9 | c.1148 C>A | p.Pro383His | Helix 10 | 71 | C |
| 9 | c.1148C>T | p.Pro383Leu | Helix 10 | 56 | B |
| 9 | c.1198C>T | p.Arg400Cys | Cytoplasmic loop 10–11 | 66 | C |
| 9 | c.1199G>A | p.Arg400His | Cytoplasmic loop 10–11 | 11 | A |
| 9 | c.1243A>G | p.Asn415Asp | Helix 11 | 17 | A |
| 9 | c.1261T>C | p.Cys421Arg | Helix 11 | 62 | B |
| 9 | c.1269delG | p.Gln423His fs*85 | Cytoplasmic loop 11–12 | 12 | A |
| 9 | c.1263-1276 del ins AGAACATGTGGAC | P.Cys 421* | Helix 11 | 78 | C |
| 10 | c.1372C >T | p.Arg458Trp | Cytoplasmic tail | 20 | A |
|  | deletion in exon 1 | / | / | 69 | C |
|  | deletions in exons 1-2 | / | / | 9 | A |
|  | deletions in exons 3-10 | / | / | 30 | A |
|  | deletions in exons 2-8 | / | / | 89 | C |
|  | deletions in exons 3-9 | / | / | 42 | A |
|  | deletions in exons 1-10 | / | / | 5 | A |
|  | entire gene deletions | / | / | 7,45 | A |
|  | entire gene deletions |  |  | 86 | C |

Phenotypes: A, early-onset classical phenotype; B, late-onset classical phenotype; C, non-classical phenotype.
